# Overexpression of Fli-1 in astrocytoma is associated with poor prognosis

**DOI:** 10.18632/oncotarget.16303

**Published:** 2017-03-16

**Authors:** Hung-Pei Tsai, Tai-Hsin Tsai, Ya-Ju Hsieh, Yi-Ting Chen, Chih-Ling Lee, Yi-Cheng Tsai, Ting-Chang She, Chih-Lung Lin, Chee-Yin Chai, Aij-Lie Kwan

**Affiliations:** ^1^ Graduate Institute of Medicine, College of Medicine, Kaohsiung Medical University, Kaohsiung, Taiwan; ^2^ Division of Neurosurgery, Department of Surgery, Kaohsiung Medical University Hospital, Kaohsiung, Taiwan; ^3^ Department of Surgery, Faculty of Medicine, College of Medicine, Kaohsiung Medical University, Kaohsiung, Taiwan; ^4^ Department of Medical Imaging and Radiological Sciences, Kaohsiung Medical University, Kaohsiung, Taiwan; ^5^ Department of Pathology, Kaohsiung Medical University Hospital, Kaohsiung, Taiwan; ^6^ Department of Pathology, College of Medicine, Kaohsiung Medical University, Kaohsiung, Taiwan; ^7^ Institute of Biomedical Sciences, National Sun Yat-Sen University, Kaohsiung, Taiwan; ^8^ Department of Neurosurgery, University of Virginia, Charlottesville, VA, USA

**Keywords:** astrocytoma, Fli-1, prognostic marker

## Abstract

**Background:**

Astrocytoma, a common and highly malignant type of brain tumor, is associated with poor overall survival despite advances in surgical treatment, radiotherapy, and chemotherapy. The nuclear transcription factor Fli-1 has been shown to increase cellular proliferation and tumorigenesis in many types of cancer; however, previous reports have not described a correlation between clinical outcomes and Fli-1 in astrocytoma patients. The present study aimed to elucidate the clinical role of Fli-1 in astrocytoma.

**Results:**

High-level of Fli-1 protein expression was significantly association with World Health Organization (WHO) high grade and poor prognosis. A multivariate analysis revealed that the WHO grade and Fli-1 protein expression were independent factor of prognostic factors of patients with astrocytoma. In addition, Fli-1 silencing inhibited proliferation, migration, and invasion and led to the downregulation of Ki-67, VEGF, and cyclin D1 expression in the astrocytoma cells.

**Materials and methods:**

Fli-1 protein expression in astrocytoma tissue samples were detected via immunohistochemistry, and potential correlations between clinical parameters and Fli-1 expression were assessed in patients with astrocytoma. Additionally, proliferation, invasion, and migration assays of astrocytoma cell lines were conducted to evaluate the effects of short interfering RNA (siRNA) on these processes; in addition, these cells were subjected to western blotting to detect the expression levels of Fli-1, Ki-67, VEGF, and Cyclin D1.

**Conclusion:**

Fli-1 shows promise as a potential prognostic biomarker and therapeutic molecular target for astrocytoma patients.

## INTRODUCTION

Astrocytoma is a common type of brain tumor in humans. According to the World Health Organization (WHO), astrocytomas can be classified into four grades [1]. Grade I astrocytomas, such as pilocytic astrocytomas, are benign and slow-growing, and grade II astrocytomas comprise relatively slow-growing diffuse tumors. However, grade III and grade IV tumors, exemplified by anaplastic astrocytoma and glioblastoma, respectively, are highly malignant. Grade III astrocytomas exhibit mitotic histopathology. Grade IV astrocytoma or glioblastoma is the most aggressive human malignant primary brain tumor and is characterized by histopathologic features, such as vascular thrombosis, microvascular proliferation, or necrosis. Despite advances in surgical treatment, radiotherapy, and chemotherapy, the overall survival of glioblastoma patients remains poor. Specifically, patients with a diagnosis of glioblastoma have the median survival period of only 12–15 months, with only 10% of the patients surviving 5 years [2] and a median survival of merely 3–4 months without treatment [3].

In humans, friend leukemia integration 1 transcription factor (Fli-1), also known as transcription factor ERGB, is encoded by the Fli-1 gene, a proto-oncogene and features a 98-amino-acid DNA binding domain [4, 5]. The Ewing sarcoma t(11;22)(q24;q12) translocation substitutes a putative RNA-binding domain of the Ewing sarcoma gene (*EWS*) on chromosome 22 for the DNA-binding domain-encoding region of *FLI-1* on chromosome 11 to yield a chimeric transcription factor that requires the DNA binding domain encoded by *FLI-1* for transformation. Fli-1, a member of the ETS transcription factor family, is also the target of insertional activation by Friend murine leukemia virus (F-MuLV) and is preferentially expressed in vascular endothelial cells and hematopoietic tissues [6]. ETS family transcription factors regulate the expression of oncogenes, tumor suppressor genes, and other genes related to vessel formation, invasion, and metastasis, and expression of these factors often correlates with poor survival [7–10].

Fli-1 affects cellular proliferation and tumorigenesis in Ewing sarcoma and primitive neuroectodermal tumors [11, 12]., and additionally plays critical roles in normal development, hematopoiesis, and oncogenesis through its dual functions as a transcriptional activator and repressor [13–17]. Previous studies have shown that knocking-down Fli-1 leads to marked growth inhibition and death in erythroleukemic cells, indicating a possible use of Fli-1 as a therapeutic target to induce tumor suppression [18–20]. Other studies identified Fli-1 overexpression as a biomarker of certain cancers including melanoma [21], ovarian cancer [22], endometrial cancer [23], breast cancer [24], and nasopharyngeal carcinoma (NPC) [25]. However, no previous studies have identified a correlation between Fli-1 protein expression and the clinical parameters associated with astrocytoma. Therefore, the present study aimed to validate the clinical role of Fli-1 in patients with astrocytoma.

## RESULTS

### Correlations between Fli-1 expression and clinical parameters

**Figure 1:**
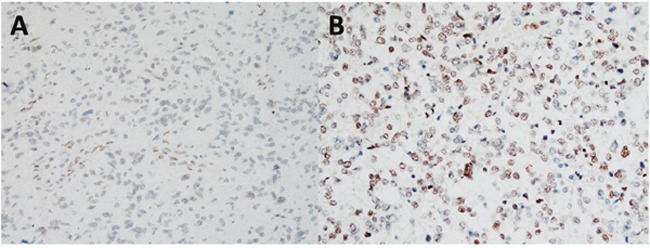
Representative results of immunohistochemical staining for Fli-1, using samples obtained from astrocytoma patients with different scores **(A)** Astrocytoma without or with weak Fli-1 expression (score: 1–3). **(B)** Astrocytoma with moderate or strong Fli-1 expression (score: 4–9). Magnification, 100X.

Of the 108 astrocytoma patients included in the study, 27 and 81 were >60 years and ≤60 years, respectively. In addition, 28 and 80 patients had grade II and III/IV astrocytoma, respectively, according to the WHO classification, and 74 and 34 cases had a Karnofsky performance score (KPS) of ≤70 and >70, respectively. Figure 1 presents examples of immunohistochemically stained sections exhibiting low and high levels of nuclear Fli-1. Chi-square analysis revealed a significant association between Fli-1 expression and the WHO grade (*P* < 0.001; Table 1).

**Table 1:** Correlation of Fli-1 expression with clinicopathologic parameters in patients with astrocytoma

	No. of patients	Fli-1 expression (n, %)		*P*-value
		Low	High	
**Age (years)**				0.801
>60	27	6 (5.6%)	21 (19.4%)	
≤60	81	22 (20.4%)	59 (54.6%)	
**Sex**				1
Male	61	16 (14.8%)	45 (41.7%)	
Female	47	12 (11.1%)	35 (32.4%)	
**WHO Grade**				<0.001
II	28	18 (16.7%)	10 (9.3%)	
III/IV	80	10 (9.3%)	70 (64.8%)	
**Tumor size**				0651
≤3cm	68	19 (17.6%)	49 (45.4%)	
>3cm	40	9 (8.3%)	31 (28.7%)	
**KPS**				0.159
≤70	74	16 (14.8%)	58 (53.7%)	
>70	34	12 (11.1%)	22 (20.4%)	

*Statistical significance (*P* < 0.05).

WHO, World Health Organization; KPS, Karnofsky performance score.

### Survival analysis

**Figure 2:**
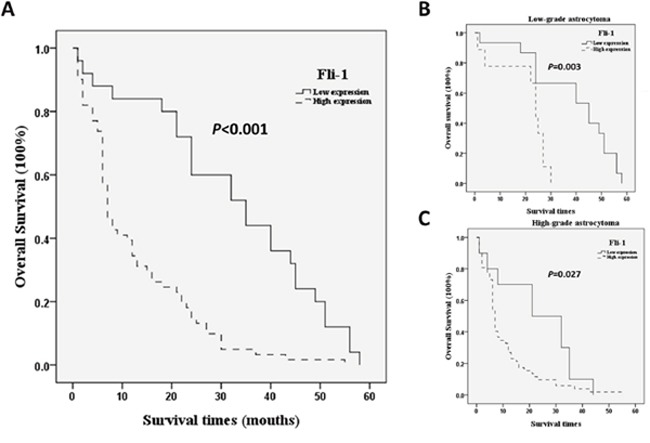
Kaplan–Meier analysis of overall patient survival according to Fli-1 expression Significance was determined using the log-rank test. **(A)** All astrocytoma patients. **(B)** Low-grade astrocytoma patients **(C)** High-grade astrocytoma patients.

A Kaplan–Meier analysis and subsequent log-rank analysis confirmed the correlation between Fli-1 expression and survival in astrocytoma patients; specifically, a high level of Fli-1 expression correlated significantly with poor overall survival (*P* < 0.001; Figure 2A). In low grade (WHO grade II) astrocytoma, a high level of Fli-1 expression correlated significantly with poor overall survival (*P* = 0.003; Figure 2B). In high grade (WHO grade III/VI) astrocytoma, a high level of Fli-1 expression correlated significantly with poor overall survival (*P* = 0.027; Figure 2C). A univariate analysis identified the WHO grade (*P* = 0.001) and Fli-1 expression (*P* = 0.001) as factors significantly associated with prognosis. The multivariate Cox regression analysis further identified the WHO grade (hazard ratio [HR], 0.475; 95% confidence interval, 0.267–0.846; *P* = 0.011) and Fli-1 protein expression (HR, 0.401; 95% confidence interval, 0.225–0.714; *P* = 0.002) as independent factors associated with prognosis in astrocytoma patients (Table 2).

**Table 2:** Univariate and multivariate Cox regression analyses of prognostic parameters in patients with astrocytoma

	Univariate analysis			Multivariate analysis		
	Relative risk	95% CI	*P*	Relative risk	95% CI	*P*
**Age**	0.866	0.503-1.492	0.605			
**Sex**	1.069	0.668-1.710	0.782			
**WHO grade**	0.365	0.203-0.656	0.001	0.475	0.267-0.846	0.011
**Tumor size**	1.253	0.776-2.024	0.357			
**KPS**	1.491	0.882-2.521	0.136			
**Fli-1 expression**	0.369	0.207-0.659	0.001	0.401	0.225-0.714	0.002

*Statistical significance (*P* < 0.05).

CI, confidence interval; WHO, World Health Organization; KPS, Karnofsky performance score.

### Elevated Fli-1 protein expression in astrocytoma cells relative to normal cells

**Figure 3:**
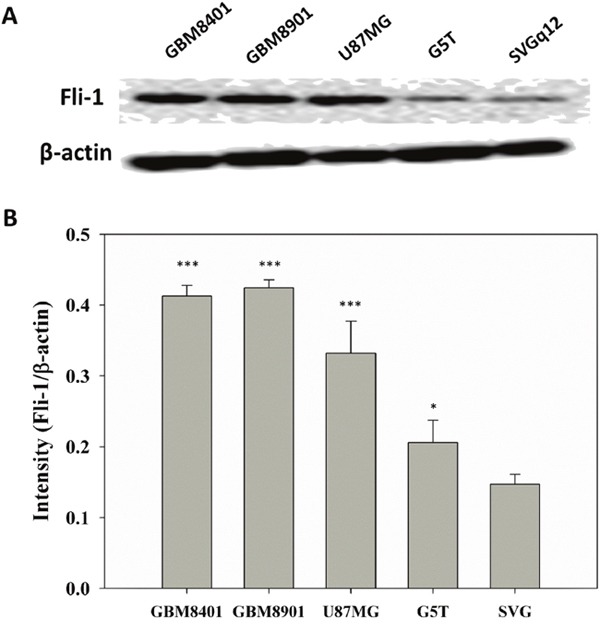
Fli-1 protein expression in all tested astrocytoma cell lines **(A)** Western blot of Fli-1. **(B)** Relative Fli-1 protein expression levels (*P < 0.05, ***P < 0.001 vs. SVGq12).

**Figure 4:**
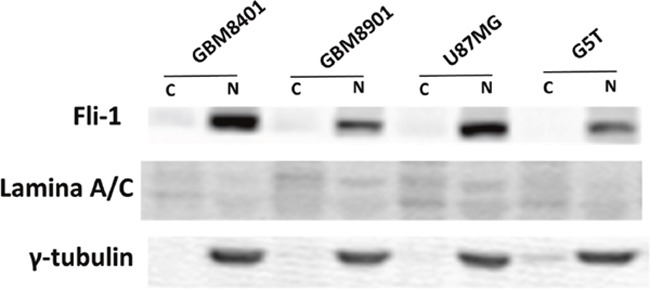
Western blots showing the Fli-1 protein of the isolated nucleus/cytoplasm sample, nuclear (N) and cytoplasmic (C)

**Figure 5:**
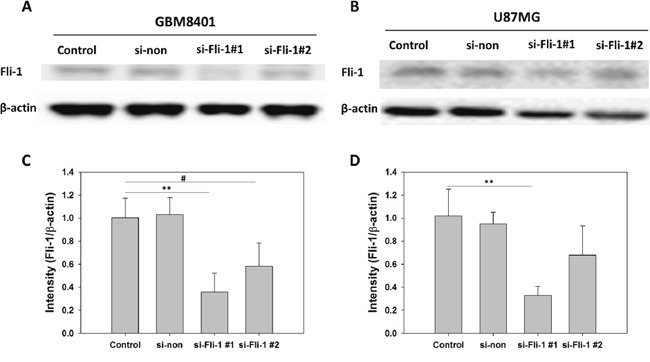
Comparison of Fli-1 expression in control, non-siRNA-treated, and Fli-1 siRNA-treated astrocytoma cells **(A)** Western blot of Fli-1 in GBM8401 cells. **(B)** Western blot of Fli-1 in U87MG cells. **(C)** Relative Fli-1 protein expression levels in GBM8401 cells. **(D)** Relative Fli-1 protein expression levels in U87MG cells (*P < 0.05, **P < 0.01, and ***P < 0.001 compared with si-Fli-1 #1; **^#^***P* < 0.05, **^##^***P* < 0.01, and **^###^***P* < 0.001 compared with si-Fli-1 #2).

The levels of Fli-1 expression in the glia cell line SVGq12 and astrocytoma cell lines GBM8401, GBM8901, U87MG, and G5T were analyzed via western blotting. GBM8401 (*P* < 0.001), GBM8401 (*P* < 0.001), U87MG (*P* < 0.001), and G5T (*P* = 0.041) expressed significantly higher levels of Fli-1 protein, compared with SVGq12 (Figure 3). The cellular localization of Fli-1 was evaluated in astrocytoma cells. Cellular fractionation was verified by the presence of Lamin A/C and γ-tubulin in the nuclear and cytoplasmic fractions, respectively. Notably, Fli-1 was present in the nuclear fraction (Figure 4). In addition, GBM8401 cells and U87MG cells were subjected to siRNA-induced knockdown of Fli-1. After a 48-h incubation with Fli-1 siRNA (si-Fli-1 group) or nonsense siRNA (si-non group), Fli-1 protein expression levels were compared between the control/si-non groups and siRNA group via western blotting. In GBM8401 cells, western blotting revealed that Fli-1 knockdown decreased protein expression of Fli-1 with si-Fli-1 #1 (*P* =0.004) or si-Fli-1 #2 (*P* =0.028) (Figure 5A and 5C). However, in U87MG cells, western blotting revealed that Fli-1 knockdown decreased protein expression of Fli-1 with si-Fli-1 #1 (*P* =0.009), but not with si-Fli-1 #2 (Figure 5B and 5D). The results indicated that Fli-1 knockdown successfully downregulated the expression of Fli-1 protein, as intended, and revealed no significant differences between the control group and si-non group.

### Fli-1 knockdown attenuated astrocytoma cell proliferation

**Figure 6:**
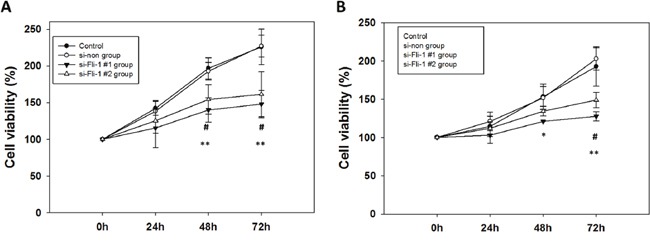
Cell viability analysis of Fli-1 siRNA-transfected (A) GBM8401 cells and (B) U87MG cells cultured for 24h, 48h, and 72h in 6-well plates (*P < 0.05, **P < 0.01, and ***P < 0.001 compared with si-Fli-1 #1; **^#^***P* < 0.05, **^##^***P* < 0.01, and **^###^***P* < 0.001 compared with si-Fli-1 #2).

To evaluate Fli-1 siRNA treatment on proliferation, we used a MTT assay to detect GBM8401 cells and U87MG cells proliferation and compared the results of the si-Fli-1. After 24-h, 48-h, and 72-h incubation with siRNA, cell viability was assayed via MTT assay. In GBM8401 cells, the results indicated reduced cell viability in the si-Fli-1 #1 (*P* =0.006) and si-Fli-1 #2 (*P* =0.035) group relative to the control on 48 hours, and the reduced cell viability in the si-Fli-1 #1 group (*P* =0.008) and si-Fli-1 #2 group (*P* =0.025) relative to the control on 72 hours (Figure 6A). In U87MG cells, the results indicated reduced cell viability in the si-Fli-1 #1 group (*P* =0.048) relative to the control on 48 hours, and the reduced cell viability in the si-Fli-1 #1 group (*P* =0.005) and si-Fli-1 #2 group (*P* =0.049) relative to the control on 72 hours (Figure 6B). However, no differences were found between the control and si-non groups in GBM8401 cells and U87MG cells. These data indicate a correlation between Fli-1 knockdown and reduced cell viability in astrocytoma cells.

### Fli-1 knockdown inhibited astrocytoma cell migration

**Figure 7:**
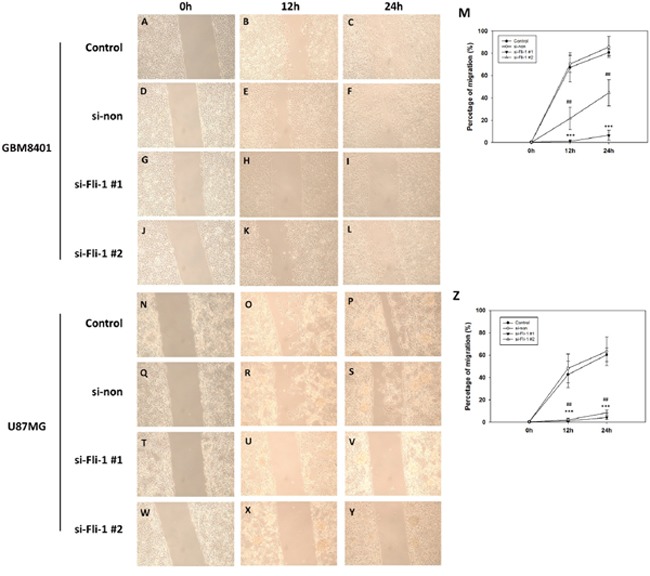
Wound healing analysis of GBM8401 cells and U87MG cells transfected with Fli-1 siRNA at 0, 12, and 24 hours after monolayer injury Representative GBM8401 cells images from the **(A–C)** control group, **(D–F)** non-siRNA group (si-non), **(G–I)** si-Fli-1 #1 group, and **(J-L)** si-Fli-1 #2 group. **(M)** The percentage of migration. Representative U87MG cells images from the **(N-P)** control group, **(Q-S)** non-siRNA group (si-non), **(T-V)** si-Fli-1 #1 group, and **(W-Y)** si-Fli-1 #2 group. **(Z)** The percentage of migration. (*P < 0.05, **P < 0.01, and ***P < 0.001 compared with si-Fli-1 #1; **^#^***P* < 0.05, **^##^***P* < 0.01, and **^###^***P* < 0.001 compared with si-Fli-1 #2).

To evaluate cell migration, we used a wound healing assay and compared between control group and si-Fli-1 group. In GBM8401 cells, si-RNA-induced Fli-1 knockdown with si-Fli1 #1 (*P* < 0.001) and si-Fli-1 #2 (*P* =0.002) markedly inhibited the migratory capability at 12 hours, and knockdown of Fli-1 with si-Fli-1 #1 (*P* < 0.001) and si-Fli-1 #2 (*P* =0.004) markedly inhibited the migratory capabilities at 24 hours (Figure 7A-7M). In U87MG cells, si-RNA-induced Fli-1 knockdown with si-Fli1 #1 (*P* =0.003) and si-Fli-1 #2 (*P* =0.004) markedly inhibited the migratory capabilities at 12 hours, and knockdown of Fli-1 with si-Fli1 #1 (*P* < 0.001) and si-Fli-1 #2 (*P* < 0.001) markedly inhibited the migratory capability at 24 hours (Figure 7N-7Z). These data suggest that knockdown of Fli-1 inhibit migration in astrocytoma cells.

### Fli-1 knockdown inhibited astrocytoma cell invasion

**Figure 8:**
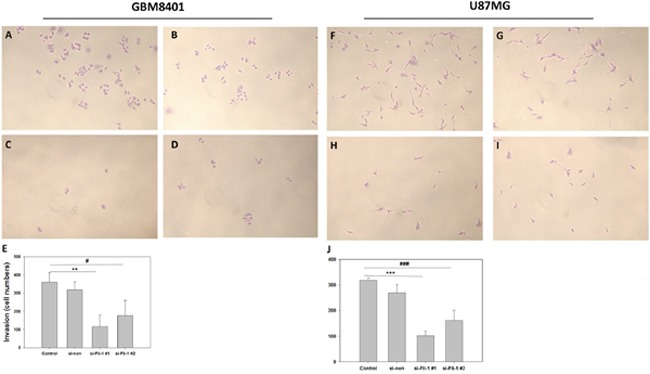
Single-day Transwell invasion analysis of GBM8401 cells and U87MG cells transfected with Fli-1 siRNA Representative GBM8401 cells images from the **(A)** control group, **(B)** non-siRNA group (si-non), **(C)** si-Fli-1 #1 group, and **(D)** si-Fli-1 #1 group. **(E)** The number of invaded cells (***P < 0.001). Representative U87MG cells images from the **(F)** control group, **(G)** non-siRNA group (si-non), **(H)** si-Fli-1 #1 group, and **(I)** si-Fli-1 #1 group. **(J)** The number of invaded cells (***P < 0.001). (*P < 0.05, **P < 0.01, and ***P < 0.001 compared with si-Fli-1 #1; **^#^***P* < 0.05, **^##^***P* < 0.01, and **^###^***P* < 0.001 compared with si-Fli-1 #2).

To evaluate cell invasion, we used a Matrigel invasion assay and compared between control group and si-Fli-1 group. In GBM8401 cells, si-RNA-induced Fli-1 knockdown with si-Fli1 #1 (*P* = 0.001) and si-Fli-1 #2 (*P* = 0.011) markedly inhibited the invasive capability (Figure 8A-8E). In U87MG cells, si-RNA-induced Fli-1 knockdown with si-Fli1 #1 (*P* < 0.001) and si-Fli-1 #2 (*P* < 0.001) markedly inhibited the invasive capability (Figure 8F-8J). These data suggest that knockdown of Fli-1 inhibit invasion in astrocytoma cells.

### Effect of Fli-1 knockdown on Ki-67, cyclin D1, and vascular endothelial growth factor (VEGF) protein expression

**Figure 9:**
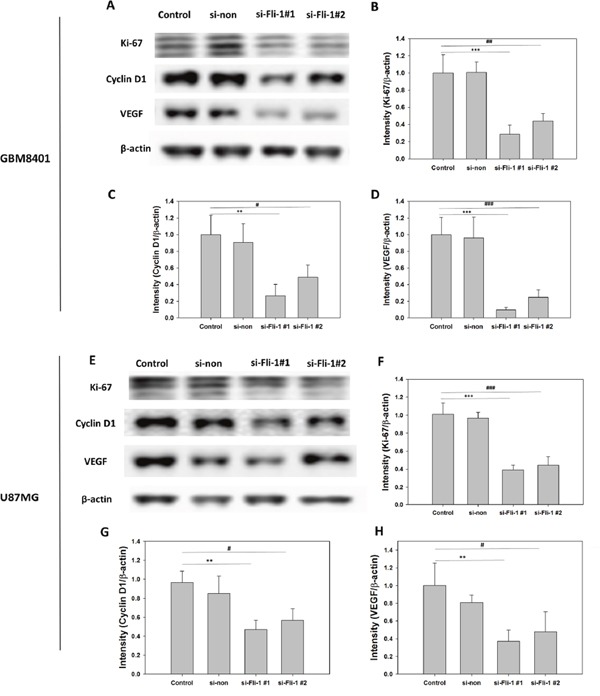
Expression levels of Ki-67, vascular endothelial growth factor (VEGF), and cyclin D1 proteins in control, non-siRNA-treated, and Fli-1 siRNA-treated cells in GBM8401 cells and U87MG cells **(A)** Western blot to assess Ki-67, VEGF, and cyclin D1 expression in GBM8401 cells. Relative protein expression levels of **(B)** Ki-67, **(C)** VEGF, and **(D)** cyclin D1 GBM8401 cells. **(E)** Western blot to assess Ki-67, VEGF, and cyclin D1 expression in U87MG cells. Relative protein expression levels of **(F)** Ki-67, **(G)** VEGF, and **(H)** cyclin D1 U87MG cells. (*P < 0.05, **P < 0.01, and ***P < 0.001 compared with si-Fli-1 #1; **^#^***P* < 0.05, **^##^***P* < 0.01, and **^###^***P* < 0.001 compared with si-Fli-1 #2).

To evaluate the expression of proteins related to cell proliferation and migration, a western blot analysis of Ki-67, VEGF, and cyclin D1 was conducted. Ki-67 and cyclin D1 are markers of astrocytoma malignancy, and VEGF is a marker of angiogenesis. In GBM8401 cells, western blotting revealed that Fli-1 knockdown led to the downregulation of Ki-67 (*P* < 0.001), cyclin D1 expression (*P* = 0.001), and VEGF expression (*P* < 0.001) with si-Fli-1 #1 and the downregulation of Ki-67 (*P* = 0.001), cyclin D1 expression (*P* = 0.019), and VEGF expression (*P* < 0.001) with si-Fli-1 #2 (Figure 9A-9D). In U87MG cells, western blotting revealed that Fli-1 knockdown led to the downregulation of Ki-67 (*P* < 0.001), cyclin D1 expression (*P* = 0.002), and VEGF expression (*P* = 0.003) with si-Fli-1 #1 and the downregulation of Ki-67 (*P* < 0.001), cyclin D1 expression (*P* = 0.011), and VEGF expression (*P* = 0.023) with si-Fli-1 #2 (Figure 9E-9H). These data suggest that knockdown of Fli-1 decreased Ki-67, cyclin D1, and VEGF protein expression in astrocytoma cells.

## DISCUSSION

The potential oncogenic nature of Fli-1 was previously demonstrated in erythroblastic leukemia, wherein Fli-1 was shown to induce the proliferation of differentiation-arrested erythroblasts with longer survival of Fli-1 expressing with BCL2 expression [26]. In a previous study of malignant melanoma, Fli-1 expression was found to be associated with a higher proliferation rate of neoplastic cells. Fli-1 expression was also detected in Merkel cell carcinoma. Fli-1 expression was negative in the control group, increased in early-stage tumors, and reached a highest level in advanced-stage tumors. Clinicopathologic analysis of Fli-1 expression revealed positive correlations of high Fli-1 expression with an advanced tumor stage and positive lymph nodal involvement. Patients expressing high levels of Fli-1 had poor overall and disease-free survival outcomes. Taken together, these findings suggest that Fli-1 is an attractive candidate for risk prognostication and targeted therapy of EOC [22]. Moreover, Fli-1 expression was associated with poorer overall survival, DMFS, and progression-free survival and was confirmed as an independent prognostic factor in a multivariate analysis of NPC [25].

In the present study, a high Fli-1 expression level was associated with poor prognosis and a high WHO grade in patients with astrocytoma; in other words, Fli-1 expression associates strongly with survival in this patient population. Increasing expression of Fli-1 is associated with tumor development and may possibly with malignancy. In a previous study, Fli-1 knockdown reduced ovarian cancer cell proliferation but did not affect tumor metastasis [22]. However, in our study, Fli-1 siRNA-mediated knockdown inhibited the proliferative, migration, and invasion abilities of astrocytoma cells.

Previous studies have reported that Fli-1 was predominantly expressed in the nuclei of Ewing sarcoma and leukemia cells [27], whereas other studies of ovarian cancer tissues and SKOV3 cells reported mainly cytoplasmic expression of Fli-1 [22]. In our study, Fli-1 was detected in the nuclei of astrocytoma tissues, as well as GBM8401, GBM8901, U87-MG, and G5T cells. These data suggest that Fli-1 plays an essential nuclear role in astrocytomas. Moreover, previous studies support a potential function of Fli-1 through protein–protein interactions or as a nuclear transcription factor involved in cellular proliferation and tumorigenesis [17, 18, 28, 29].

The role of Fli-1 has been investigated in Ewing sarcoma and primitive neuroectodermal tumors; this potential role is implicated by the presence of a specific translocation t(11;22) that resulting in a fusion of *EWS* on chromosome 22 to *FLI1* on chromosome 11 in 90% of cases of these tumors [11, 12]. Further speculation suggests that Fli-1 is widely expressed in various cancer tissues but plays different, tissue-specific roles. For example, angiogenesis is required to provide proliferating tumor cells with nutrients and metabolites, and tumor cells regulate angiogenesis by secreting factors that stimulate neovascularization. Epithelial cells have been reported to express high levels of Fli-1 [30], a feature that has also been observed in the majority of benign and malignant vascular tumors [31–33]. The EWS-Fli-1 fusion protein directly activates VEGF-A, leading to increased angiogenesis and malignant progression [34]. High-levels of VEGF, which plays a critical role in tumor initiation, have also been detected in the tumor microenvironments of Fli-1-overexpressing erythroleukemias [35]. These results indicate the potential usefulness of Fli-1 as a biomarker of angiogenesis.

Previous studies have defined a role for Fli-1 in erythroleukemic cell growth and differentiation. One such study demonstrated the binding of Fli-1 to an ETS consensus site in the context of cell growth maintenance [36]. ETS family transcription factors regulate the expression of oncogenes, tumor suppressor genes, and other genes associated with vessel formation, invasion, and metastasis, and their expression often correlates with poor survival in some types of cancers [21, 37–39]. Fli-1 plays a critical role in normal development, hematopoiesis and oncogenesis by functioning as both transcriptional activator and repressor [34, 40, 41]. Knocking-down Fli-1 expression in erythroleukemic cells leads to a marked growth inhibition and cell death, demonstrating a possible therapeutic approach to induce tumor suppression [42, 43]. The observation of strong Fli-1 expression in adenoid-like differentiated NPC suggests that NPC cancer cells might develop adenoid-like endothelium consequent to Fli-1-mediated gene expression. The EWS/FLI-1 fusion product promotes tumor angiogenesis by upregulating VEGF-A expression [34], and in our study, Fli-1 knockdown attenuated the expression of Ki-67, VEGF, and cyclin D1 proteins. These data supported a role for Fli-1 in the regulation of tumor progression.

## MATERIALS AND METHODS

### Patients

For this study, we selected patients with astrocytoma who were treated at the Neurosurgery Department of Chung Ho Hospital, Kaohsiung Medical University between 2002 and 2014. Patients who were diagnosed by biopsy only or had incomplete medical records, no follow-up data, low-quality pathological results, or poor immunohistochemical staining were excluded. A total of 109 patients were selected for this study.

### Immunohistochemical staining

Three-micrometer sections were cut from formalin-fixed, paraffin-embedded tissue samples collected from each patient. Sections were deparaffinized, rehydrated, and autoclaved at 121°C for 10 min in Target Retrieval solution, pH 6.0 (S2369; Dako, Glostrup, Denmark) for antigen retrieval. After letting the sections rest for 20 min at room temperature, 3% hydrogen peroxide was applied for 5 min at room temperature to block endogenous peroxidase. After washing twice with Tris buffer, the sections were incubated with a Fli-1 antibody (1:200 dilution) for 1 hour at room temperature. After washing twice with Tris buffer, the sections were incubated with a secondary horseradish peroxidase-conjugated antibody for 30 min at room temperature. Finally, the slides were incubated in 3,3-diaminobenzidine (K5007; Dako) for 5 min, counterstained with Mayer's hematoxylin for 90 sec, and mounted with Malinol.

Immunohistochemical staining results were classified as low-level expression and high-level expression. Scores, which represented the proportions of positively stained tumor cells, were determined as follows: 0, no positive tumor cells; 1, <10% positive cells; 2, 10–50% positive cells; and 3, >50% positive cells. The staining intensity was classified as 0, no staining; 1, weak staining; 2, moderate staining; or 3, strong staining. The staining index (SI) was calculated by multiplying the intensity and percentage of positive tumor cells in each sample to yield possible scores of 0, 1, 2, 3, 4, 6, and 9. We set a total score of 4 as a cut-off; in other words, ≥4 was considered high Fli-1 expression, and <3 was considered low expression.

### Cell culture and Fli-1 siRNA transfection

All cell lines were incubated at 37°C in an atmosphere of 5% CO_2_. The cell lines GBM8401 and GB8901 were cultured in RPMI medium supplemented with 10% fetal bovine serum (FBS). U87-MG and SVGp12 were cultured in modified Eagle's medium (MEM) supplemented with 10% FBS. G5T was cultured in Dulbecco's MEM (DMEM) medium supplemented 10% FBS. GBM8401, GBM8901, U87-MG, and G5T were isolated from GBM patients; SVGp12 was isolated from normal tissue and used as a normal control.

siRNA transfection of astrocytoma cells was achieved using DharmaFECT™ Transfection Reagents (Dharmacon, Lafayette, CO, USA) and human Fli-1 siRNA constructs (Sigma, St. Louis, MO, USA) with the following sequences: Fli-1 siRNA#1 5′-GUUCACUGCUGGCCUAUAA-3′ and; Fli-1 siRNA#2 5′-CACAAACGAUCAGUAACAA-3′. A 5-μM Fli-1 siRNA concentration was used for transfection. Following transfection with siRNA, cells were cultured for 1 days before use. Fli-1 protein expression levels were detected by western blot analysis.

### Proliferation assay

Cells were suspended in culture medium containing 10% FBS and placed in a 24-well plate at an approximate density of 1 × 10^4^ cells per 0.5 ml of medium per each well. The cells were incubated as described above for 24 h, 48h, and 72h with 3-(4,5-dimethylthiazol-2-yl)-2,5-diphenyltetrazolium bromide (MTT) assay.

### Migration assay

Cell migration was evaluated using a wound healing assay (80209; ibidi GmbH, Martinsried, Germany). For this assay, 6-well plates were coated with culture-inserts and incubated at 37°C for 12 h. Cells were seeded 70μl at a density of 5 × 10^5^ cell/ml and incubated for 12 hours and 24 hours with 10μg/ml mitomycin C (Sigma) after 48 hours prior to siRNA transfection.

### Invasion assay

*In vitro* cell invasion assays were performed using Transwell chambers (COR3452; CORNING, Corning, NY, USA). Cells after siRNA transfection were seeded at a density of 1,000 cells per insert, and the lower chamber of each Transwell was filled with 2 ml of medium containing 10%FBS. After a 24-h incubation, cells remaining on the upper surfaces of the Transwell membranes were removed using cotton swabs. Cells that had invaded across the membranes to the bottom of the insert were fixed, stained, photographed, and quantified by counting the numbers of cells in 6 random high-powered fields.

### Cytoplasmic and nuclear protein extraction

Cells were detached from culture plates using trypsin, and centrifuged at 500 g for 3 min to obtain cell pellets. Each pellet was mixed with 200 μl of CER, mixed, and vortexed at a high speed for 15 s, followed by a 30-min incubation on ice. The mixture was vortexed again at a high speed for 5 s and centrifuged at 16000 g for 10 min at 4°C, after which the supernatant was removed and reserved as the cytoplasmic protein fraction. Next, 50 μl of NER was added to each pellet, followed by thorough high-speed vortexing for 15 seconds and incubation on ice for 30 minutes. During this incubation, the mixtures were vortexed for 20 s at a high speed every 5 min, followed by centrifugation at 16000 g for 30 mins at 4°C. The resulting supernatant was reserved as the nuclear protein fraction.

### Western blotting

All samples were lysed in 200 μl of lysis buffer. A total of 50 μg of protein per sample was loaded into the wells of a sodium dodecyl sulfate-polyacrylamide gel and subjected to electrophoresis at 50 V for 4 h. The separated proteins were subsequently transferred to PVDF membranes. After incubation for 1 h in blocking buffer, the membranes were incubated with primary antibodies [Fli-1 (554266, 1:500; BD Pharmingen, San Diego, CA, USA), Ki-67 (1:1000; Dako), cyclin D1 (RM-9104, 1:500; Thermo, UK), and β-actin (60008-1-lg, 1:20,000; proteintech, USA)] for 2 h at room temperature. Subsequently, the membranes were incubated with secondary antibodies [goat anti-rabbit (AP132P, 1:5000; Millipore, Billerica, MA, USA) and goat anti-mouse (AP124P, 1:5000; Millipore)] for 90 min. Enhanced chemiluminescence solution (Western Lightning, 205-14621; Perkin Elmer, Waltham, MA, USA) and a MiniChemi™ imaging and analysis system (Beijing Sage Creation, Beijing, China) were used to detect specific protein bands.

### Data analysis

SPSS 19.0 software (SPSS, Inc., Chicago, IL, USA) was used for the statistical analysis. The chi-square test was performed to determine the existence of correlations between Sp1 protein expression and specific clinicopathologic parameters. The Kaplan–Meier method and log-rank test were used for the survival rate analysis. Multivariate Cox regression analyses were used to verify the independent effect of each variable evaluated in this study. A one-way analysis of variance (ANOVA) was used to compare the results of the proliferation, migration, and invasion assays. For all analyses, a *p* value < 0.05 was considered statistically significant.

## CONCLUSION

In summary, Fli-1 was expressed in the nuclei of astrocytoma cells. A high level of Fli-1 expression was detected in high-grade astrocytomas and was associated with poor prognosis, and both the univariate and multivariate analyses identified an association of Fli-1 with the overall survival duration. Fli-1 knockdown attenuated the proliferation, migration, and invasion of astrocytoma cells and downregulated the expression of Ki-67, VEGF, and cyclin D1. These results suggest that the potential use of Fli-1 as a prognostic biomarker in patients with astrocytoma.
